# Quercetin Decreases Corneal Haze In Vivo and Influences Gene Expression of TGF-Beta Mediators In Vitro

**DOI:** 10.3390/metabo12070626

**Published:** 2022-07-07

**Authors:** Tina B. McKay, Pouriska B. Kivanany, Sarah E. Nicholas, Okhil K. Nag, Michael H. Elliott, W. Matthew Petroll, Dimitrios Karamichos

**Affiliations:** 1 Department of Cell Biology, University of Oklahoma Health Sciences Center, Oklahoma City, OK 73104, USA; tmckay@mgh.harvard.edu; 2 Department of Ophthalmology, University of Texas Southwestern Medical Center, Dallas, TX 75390, USA; pouriskakivanany@gmail.com (P.B.K.); matthew.petroll@utsouthwestern.edu (W.M.P.); 3 North Texas Eye Research Institute, University of North Texas Health Science Center, Fort Worth, TX 76107, USA; sarah.nicholas@unthsc.edu; 4 Department of Ophthalmology, Dean McGee Eye Institute, University of Oklahoma Health Sciences Center, Oklahoma City, OK 73104, USA; okhil.nag@nrl.navy.mil (O.K.N.); michael-elliott@ouhsc.edu (M.H.E.); 5 Department of Pharmaceutical Sciences, University of North Texas Health Science Center, Fort Worth, TX 76107, USA; 6 Department of Pharmacology and Neuroscience, University of North Texas Health Science Center, Fort Worth, TX 76107, USA

**Keywords:** quercetin, corneal wound healing, transforming growth factor-beta, corneal debridement, keratectomy, antifibrotic, flavonoid

## Abstract

We have previously reported the flavonoid, quercetin, as a metabolic regulator and inhibitor of myofibroblast differentiation in vitro. Our current study evaluated the effects of topical application of quercetin on corneal scar development using two different animal models followed by RNA analysis in vitro. Wild-type C57BL/6J mice were anesthetized and the corneal epithelium and stroma were manually debrided, followed by quercetin (0.5, 1, 5, or 50 mM) or vehicle application. Corneal scarring was assessed for 3 weeks by slit lamp imaging and clinically scored. In a separate animal study, six New Zealand White rabbits underwent lamellar keratectomy surgery, followed by treatment with 5 mM quercetin or vehicle twice daily for three days. Stromal backscattering was assessed at week 3 by in vivo confocal microscopy. In mice, a single dose of 5 mM quercetin reduced corneal scar formation. In rabbits, stromal backscattering was substantially lower in two out of three animals in the quercetin-treated group. In vitro studies of human corneal fibroblasts showed that quercetin modulated select factors of the transforming growth factor-β (TGF-β) signaling pathway. These results provide evidence that quercetin may inhibit corneal scarring. Further studies in a larger cohort are required to validate the efficacy and safety of quercetin for clinical applications.

## 1. Introduction

Corneal opacification affects over 1 million people worldwide and remains a major cause of worldwide blindness [1]. Loss of transparency and the development of a corneal scar (haze) is a mechanism of disruption in the highly organized collagen lamellae present in the corneal stroma. Previous studies have shown that a loss in transparency correlates with the formation of myofibroblasts and increased expression of α-smooth muscle actin that occurs in concert with decreased crystallin protein expression in the corneal stroma [2,3,4]. To-date, corneal transplantation remains the only Food and Drug Administration-approved intervention for recovering vision loss due to corneal scarring.

Quercetin and its glycosylated forms are flavonoids highly abundant in numerous food products, including apples, onions, and kale. Studies have suggested that quercetin promotes significant effects on metabolism [5,6] and inhibits lactate transport in cancer cells (HeLa and glioma cell lines) [7,8]. Its metabolic properties also extend to lipid and eicosanoid regulation, with anti-inflammatory and anti-thrombotic effects reported [9,10,11]. We have previously identified quercetin as a potent metabolic regulator that downregulates the production of lactate by corneal fibroblasts and significantly modulates the abundance of tricarboxylic acid cycle intermediates [12]. We have also found that quercetin inhibits the expression of the fibrotic markers α-smooth muscle actin and collagen type 3 in human corneal fibroblasts cultured in vitro, suggesting its therapeutic potential as an anti-scarring molecule [13]. As a natural product that is highly abundant in the human diet, therapeutic applications of quercetin in its pure form are advantageous, given its relatively safe drug profile and low toxicity in vivo [14]. However, to the authors’ knowledge, ocular applications of topically-applied, high-dose quercetin solutions on corneal wound healing have yet not been investigated.

Here, we tested the hypothesis that quercetin is a potent anti-fibrotic that may prevent scar formation following corneal wounding. Our study evaluated the effects of increasing concentrations of quercetin solutions on corneal haze in separate mouse and rabbit models for corneal wound healing. We also investigated the major effects of quercetin solutions on gene expression patterns in human corneal fibroblasts cultured in a 3D system. Our study provides evidence that the topical application of quercetin may inhibit corneal fibrosis in vivo.

## 2. Results

**Figure 1:**
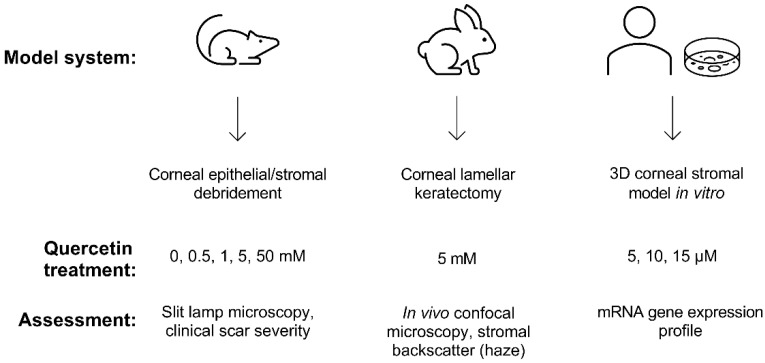
**Experimental scheme developed to assess the effects of quercetin using both in vivo and in vitro models.** Wild-type C57BL/6J mice and New Zealand white rabbit models were used to observe scar development following topical quercetin or vehicle application. The 3D corneal stromal model was generated using primary human corneal fibroblasts cultured for 4 weeks in vitro to promote the deposition of a self-assembled stromal matrix.

Building upon our previous identification of quercetin as a potent anti-fibrotic and metabolic regulator in vitro [12,13], we developed a multi-prong approach in this study to evaluate the effects of quercetin on the cornea using two different animal models (mouse and rabbit) and 3D constructs generated by primary human corneal fibroblasts cultured in vitro (Figure 1).

**Figure 2:**
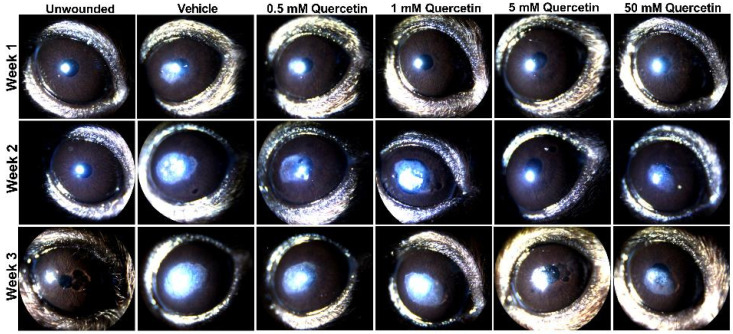
**Slit lamp images of mouse corneas at weeks 1, 2, and 3 following corneal debridement.** Four concentrations of quercetin (0.5, 1, 5, and 50 mM) in 10% DMSO (vehicle) were tested and topically applied to the cornea immediately post-debridement. Representative images shown for each treatment group (n = 4 eyes for each quercetin concentration, n = 2 unwounded eyes, and n = 2 eyes with vehicle-treatment in this experiment). Previous studies evaluating vehicle effects (n = 3) and a lower dose of quercetin treatment (50 μm, n = 3) are shown in the Supplemental Materials.

To determine if quercetin application influenced scar development, we utilized an epithelial-stromal debridement mouse model [15] to assess corneal haze development following injury. Based on slit lamp microscopy, we observed significant corneal scarring in the vehicle control (10% dimethylsulfoxide (DMSO)) in the central cornea by week 1 post-surgery that increased in size and opacity from week 2 and 3 compared to the unwounded eyes (Figure 2). The lowest concentrations of quercetin tested in this experiment (0.5 and 1 mM) showed similar scar formation as the vehicle-treated group. Mice treated with higher concentrations of quercetin (5 and 50 mM) showed a reduction in both the size and density of corneal haze as observed by slit lamp microscopy (Figure 2).

**Figure 3:**
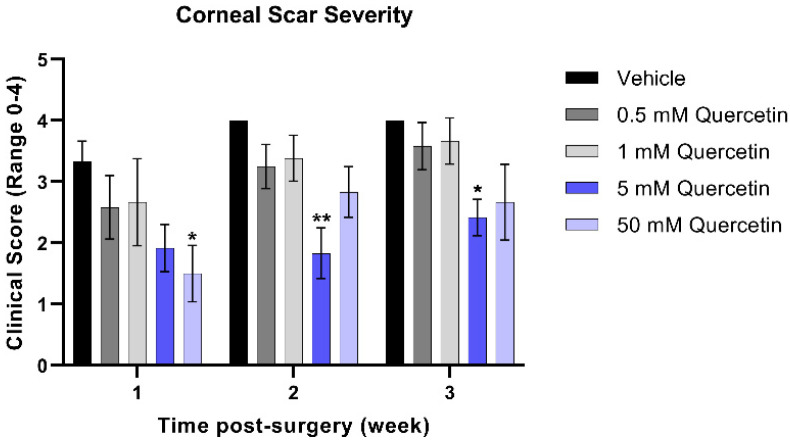
**Clinical scoring of corneal scar severity.** Quercetin-treated groups were compared to the vehicle-treated group at the same-timepoint. Statistical significance based on a two-way ANOVA with Šídák’s multiple comparisons test (* *p* < 0.05 and ** *p* < 0.01). Bars represent mean ± standard error of the mean with n = 3 clinical scores per eye.

To quantify the severity of the corneal scars that developed from weeks 1 through 3 post-injury in the mouse model, clinical scoring of corneal haze was performed by trained staff blinded to treatment groups. At the week 1 timepoint, the higher concentration (50 mM quercetin) was associated with a significant 2.2-fold reduction in scar severity compared to the vehicle-treated group (mean difference = 1.8, 95% confidence interval (CI) [0.2663 to 3.400], *p* = 0.017, Figure 3). While both high-dose quercetin-treated groups initially showed a modest reduction in scar severity, only the 5 mM quercetin-treated mice maintained a significantly 2.2-fold lower scar severity at week 2 compared to the vehicle-treated controls (mean difference = 2.17, 95% CI [0.5997 to 3.734], *p* = 0.004) that was maintained up to week 3 (1.7-fold, mean difference = 1.58, 95% CI [0.01634 to 3.150], *p* = 0.047). It is unclear whether quercetin treatment reduces scar development only at the initial stages or halts scar formation past 3 weeks.

**Figure 4:**
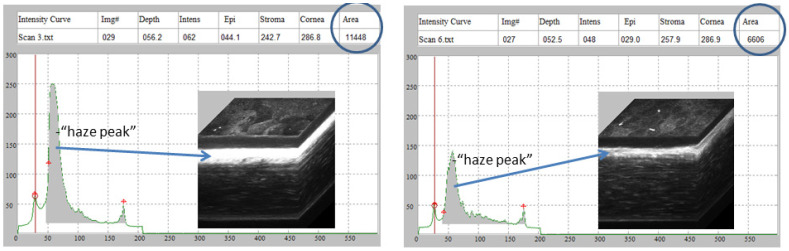
**Rabbit CMTF haze assessment.** Representative in vivo confocal microscopy through focusing (CMTF) scans and 3D reconstructions from rabbits treated with vehicle (**left**) or quercetin (**right**), 21 days after LKsurgery. The arrows denote the region of the highest haze detected within the corneal stroma.

**Figure 5:**
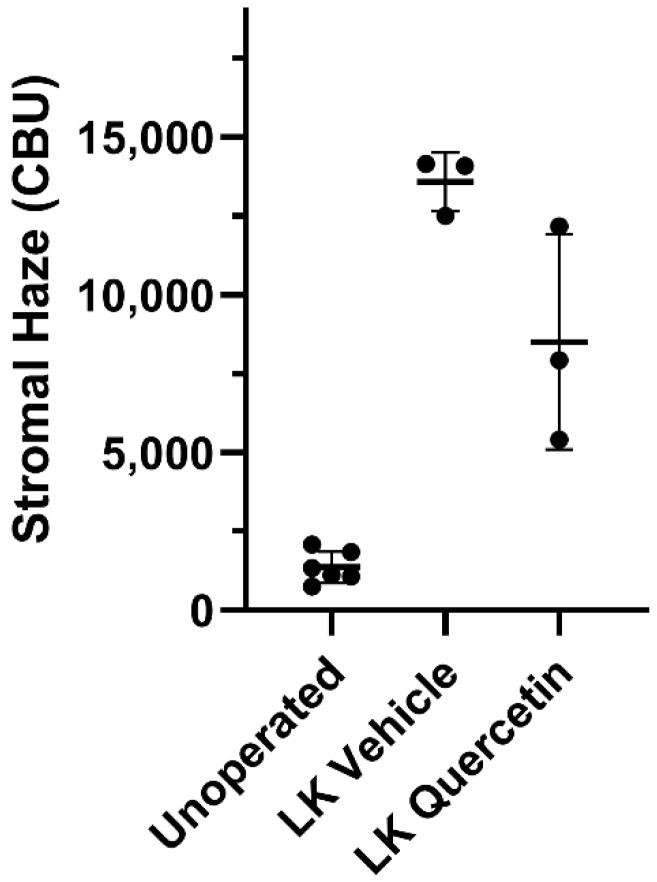
**Effect of quercetin on corneal haze.** LK induced a significant increase in corneal stromal backscatter (haze) in corneas treated with vehicle alone. In two of the three rabbits, a decrease in corneal haze was detected in the quercetin-treated group as compared to control rabbits (vehicle-treated), but this difference was not statistically significant (Brown-Forsythe ANOVA test and Dunnett’s T3 multiple comparison test). CBU = confocal backscatter units.

To assess the effect of quercetin on the development of corneal fibrosis in the rabbit model, lamellar keratectomy (LK) was performed and a quercetin or vehicle solution was applied to the corneal surface twice daily for 3 days. Previous studies have shown that peak fibrosis is observed at approximately 21 days after surgery in this model [16]. Based on our findings in the mouse model testing a concentration series of quercetin, we utilized the optimal concentration of 5 mM quercetin solution in the rabbit studies. Figure 4 shows representative scans and 3D reconstructions from rabbits treated with vehicle (left) or quercetin (right) at this time point. In two of the three rabbits treated with quercetin, a substantial decrease in stromal backscattering (haze = area under curve) was detected as compared to control rabbits (Figure 5). However, because of the variability in the response and the low sample size, the difference in haze between quercetin-treated and control animals did not reach statistical significance.

**Figure 6:**
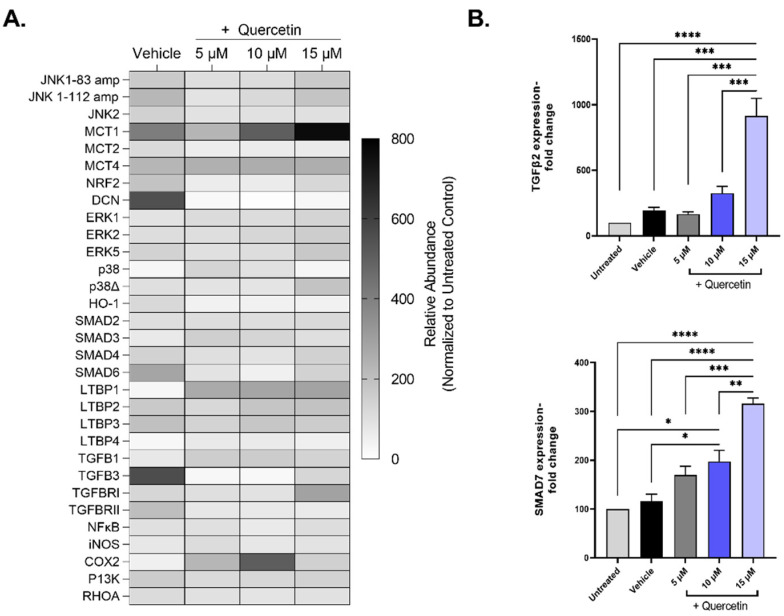
Expression of select mRNA transcripts by primary human corneal fibroblasts in 3D constructs treated with increasing concentrations of quercetin compared to untreated and vehicle-treated cultures. (**A**) Heat map of relative gene expression detected using qRT-PCR analysis. Expression was normalized to the untreated control and presented as a percentage (untreated control set at 100%). (**B**) Select genes influenced by quercetin treatment: transforming growth factor-β2 (TGFB2) mothers and against decapentaplegic homolog 7 (SMAD7). Statistical significance determined based on an ordinary one-way ANOVA analysis with Šídák’s multiple comparisons test. Bars represent mean ± standard error of the mean with n = 3. * *p* < 0.05, ** *p* < 0.01, *** *p* < 0.005, and **** *p* < 0.0001.

To investigate the mechanistic details of quercetin, we evaluated the gene expression patterns of pathways associated with extracellular matrix deposition (TGFB), cell proliferation (ERK, JNK), and lactate transporters (MCT), among others, in quercetin-treated 3D cultures of human corneal fibroblasts (Figure 6A). Of the 33 genes that showed expression in all groups, only two genes were differentially regulated by quercetin treatment independently of the vehicle: TGF-β2 (TGFB2, ↑4.8-fold in the 15 μM-treated group, *p* < 0.0001) and mothers against decapentaplegic homolog 7 (SMAD7, ↑2.7-fold in the 15 μM-treated group, *p* = 0.019; Figure 6B). Both TGF-β2 and SMAD7 are important factors involved in the TGF-β pathway. We also identified three genes that showed significant upregulation by the vehicle (DMSO) alone compared to untreated constructs, including transforming growth factor-beta 3 (TGF-β3; TGFB3, ↑5.6-fold, *p* < 0.0001), mothers against decapentaplegic homolog 6 (SMAD6, ↑2.8-fold, *p* = 0.04), and decorin (DCN, ↑5.5-fold, *p* < 0.0001).

## 3. Discussion

A number of polyphenols, including quercetin, are thought to be produced by plants in response to environmental stressors that may limit energy bioavailability, serving as a biochemical signal to other organisms of potential danger [17,18]. The therapeutic properties of flavonoids may be at least partially attributed to their linked expression to acute nutritional or environmental stress in plants that appear to activate pro-reparative responses in animals. Whether the bioactivity associated with quercetin and other similar flavonoids extends to promoting corneal regeneration remains unclear.

In this study, we found a significant reduction in corneal scar severity in mice treated with a single-drop of 5 mM quercetin immediately following injury, with the therapeutic benefit extending up to week 3. While two of the three rabbits showed substantially lower corneal haze based on the stromal backscatter detected using in vivo confocal microscopy, the results did not reach our threshold for statistical significance (*p* < 0.05). RNA analysis showed significant changes in the regulation of genes related to the TGF-β pathway, notably the upregulation of TGFB2 and the inhibitory regulator SMAD7. Increasing the expression of SMAD7 has been previously reported to blunt corneal scar development in mouse models [19,20], and thus the anti-fibrotic properties of quercetin observed in our study may be related to this significant increase in SMAD7 promoted by quercetin treatment. Elucidating the mechanisms underlying the differential regulation of the TGF-β isoforms and their respective receptors following quercetin stimulation requires further studies, including protein validation.

In terms of a role for metabolic regulation in promoting anti-fibrotic processes, quercetin has been reported as a potent inhibitor of the lactate transporter MCT1 [21,22]. We have previously identified that quercetin decreases lactate production and significantly modulates levels of both glycolysis and the tricarboxylic acid cycle [12,13]. Our results showed a modest increasing trend in gene expression levels of MCT1 with quercetin application, which may be a secondary compensatory mechanism related to its direct inhibitory effects on MCT1 function. Of note, the vehicle used in our studies, DMSO, also contributed to significant changes in the regulation of a number of genes in the absence of quercetin, including TGFB3, SMAD6, and the extracellular matrix protein decorin—which is likely a confounding factor in the in vivo wounding models as well. Given that quercetin is a highly lipophilic molecule, the development and utilization of optimal delivery mechanisms with minimal vehicle-associated side effects is required for further clinical applications of quercetin. Moreover, evaluation of these pathways following quercetin treatment in vivo are needed.

An alternative hypothesis for the findings associated with quercetin treatment in our study may be related to its known regulatory function of members of the sirtuin family of proteins, consistent with a metabolic role in tissue regeneration. Quercetin and related polyphenols are potent activators of Sirtuin 1, a nicotinamide-dependent histone deacetylase that regulates cell survival via the p53-mediated pathway [23]. Given their regenerative effects, activation of the sirtuin proteins have been proposed for the treatment of age-related diseases, including type 2 diabetes mellitus [24], cancer [25,26], and neurodegeneration [27], as well as for extending organismal lifespans [28,29]. Although evidence has been reported regarding decreased sirtuin expression and pro-fibrotic processes in various tissues [30,31], it remains unclear if sirtuin activation—specifically Sirtuin 1—via stimulation by quercetin or other similar flavonoids can inhibit corneal fibrosis in vivo. We posit that the multifaceted effects of quercetin might affect other pathways in addition to the TGF-β pathway that may be important in the anti-scarring effects seen in vivo.

Our data suggests that quercetin application following wounding may blunt scar development in the cornea. Further investigations into the mechanism of this action are required to determine if the bioactivity of pure-form quercetin is dependent on the regulation of the TGF-β pathway or other pathways similarly important in tissue regeneration—including targeted activation of members of the sirtuin family. A strength of the current study is the utilization of two different small animal model organisms (Mus musculus and Oryctolagus cuniculus) to evaluate inter-species differences and effects of different surgical approaches to evaluate any therapeutic effects of topical quercetin application. We have previously studied quercetin in vitro and found optimal concentrations to be in the micromolar range [12], while in vivo studies required a much higher concentration in the millimolar range to exhibit a therapeutic effect. We also tested multiple drug concentrations in the mouse model to identify an optimal dose (5 mM), which was utilized in these pilot rabbit studies. Given our encouraging initial results, additional optimization of the concentration and time-course of dosing is warranted for the rabbit model. In addition, studies using photorefractive keratectomy, which is more reproducible than lamellar keratectomy might also reduce the variability in response. Other limitations of the current study include the relatively small sample size (n = 3–4 eyes per quercetin treatment), the utilization of DMSO as the vehicle, and the minimal characterization of the corneas post-injury. Of note, given the low solubility of quercetin in water-based solvents, the development and optimization of safe carriers for these highly lipophilic, bioactive compounds are needed for further clinical applications. Moreover, the relative absorption and uptake of quercetin in the stroma by resident keratocytes or fibroblasts is required to determine bioavailability and optimal dosing, including reducing off-target effects. Further studies using a larger sample size and the evaluation of myofibroblast formation and collagen organization in the stroma with and without quercetin treatment are required to validate consistent anti-fibrotic effects of quercetin application following corneal wound healing.

## 4. Materials and Methods

### 4.1. Chemicals

All chemicals were purchased from Sigma Aldrich (St. Louis, MO, USA), unless otherwise noted.

### 4.2. Preparation of Quercetin Solutions

Quercetin (3,3′,4′,5,7-pentahydroxyflavone; IUPAC Name 2-(3,4-dihydroxyphenyl)-3,5,7-trihydroxychromen-4-one; CAS # 117-39-5) solutions were freshly prepared by dissolving the yellow powder in sterile DMSO, which was then vortexed and incubated for at least 10 min at room temperature in the dark to permit complete dissolution. Solutions were then diluted to their final concentrations (in vivo: 0.5, 1, 5, and 50 mM; in vitro: 5 μM, 10 μM, and 15 μM) in phosphate-buffered saline (PBS) to a final concentration of 10% DMSO, followed by filter-sterilization (0.2 μM filter).

### 4.3. Animals

All animal studies were conducted in accordance with the ARVO Statement for the Use of Animals in Ophthalmic and Vision Research. All mouse studies were approved by the Institutional Animal Care and Use Committee at the University of Oklahoma Health Sciences Center (IACUC protocol # 12-145-H). Rabbit studies were approved by the IACUC of the University of Texas Southwestern Medical Center (IACUC protocol #101490-USDA). Wild-type C57BL/6J mice were purchased from The Jackson Laboratory (Bar Harbor, ME) and maintained in the vivarium at the Dean A. McGee Eye Institute (Oklahoma City, OK, USA). Mice were anesthetized using ketamine (80 mg/kg)/xylazine (5 mg/kg) injected intraperitoneally, and a drop of proparacaine hydrochloride ophthalmic solution (0.5%) was added to the eye immediately before surgery. A 1.5 mm trephine was used to demarcate the central cornea [15]. An Algerbrush II with a rotating 0.5 mm burr was then applied to the marked region in a circular manner for 30 s to remove 1.5 mm diameter of the corneal surface, as visualized using a stereo microscope. Following debridement, 10 μL of the quercetin solution, vehicle (10% DMSO), or PBS were topically applied to the cornea while the eye was maintained in a proptosed position for a single-treatment. The eyes were imaged by slit lamp microscopy at weeks 1, 2, and 3 post-injury.

Six New Zealand white rabbits were anesthetized using 50 mg/kg intramuscular ketamine and 5.0 mg/kg xylazine and locally anesthetized in the left eye with 1 drop of proparacaine. Lamellar keratectomy (LK) was performed on the left eye of each animal. A speculum was placed into the eye and a 150 µm deep incision was made in the peripheral cornea using a diamond knife. A spatula was then used to create a resection plane by separating the layers of collagen within the stroma. Once a resection plane was created, a 5 mm circular trephine was used to remove the anterior corneal tissue in the central cornea. Immediately following surgery, 0.3 mg/kg of buprenorphine SR (slow release) was injected. Gentamicin eye drops were administered in the left eye twice a day for 7 days following surgery. Left eyes were treated topically with either 5 mM quercetin or vehicle twice daily for three days after surgery.

### 4.4. Clinical Scoring of Scar Severity

Slit lamp images of the mice were randomized and scored by three clinicians in a blinded manner, with no knowledge of treatment groups. The scoring criteria were defined as follows: (1) Score = 1.0, <1.5 mm trace haze and not in the line of sight; (2) Score = 2.0, 1.5–2.5 mm total size, easily noticeable, and approaching the line of sight; (3) Score = 3.0, >2.5 mm total size, dense but translucent haze and impinging on the line of sight; (4) Score = 4.0, >2.5 mm total size, dense and opaque haze in the line of sight.

### 4.5. In Vivo Confocal Microscopy

Rabbits were imaged at 7 and 21 days after LK using an HRT-RCM in vivo confocal microscopy with Confocal Microscopy Through Focusing (CMTF) software for analysis, as previously described [32,33]. Rabbits were anesthetized prior to scanning with 50 mg/kg intramuscular ketamine and 5.0 mg/kg xylazine and locally anesthetized in each eye with 1 drop of proparacaine. CMTF scans were collected by starting the scan in the anterior chamber and finishing above the epithelium, with a constant speed of 60 µm per second. Each scan was conducted using a gain of 6, which was set by unchecking the “auto brightness” box in the software and moving the horizontal slider six mouse clicks to the right. At least 3 scans were collected within two regions in the central area of the cornea where the LK was performed (total of at least 6 scans). Additional scans were collected in the central cornea of the unoperated contralateral eyes. Each scan contained a 3D stack of 384 × 384-pixel images (400 × 400 µm), with a step size of approximately 2 µm between images.

After image acquisition, scans were saved as “.vol” files, which could be opened by our in-house CMTF software to analyze the 3D changes in cell morphology and cell/extracellular matrix reflectivity [32]. The program generates an intensity vs. depth curve, corresponding to the average pixel intensity of each image and the z-depth of that image within the scan, respectively. The relative amount of backscatter, or haze, associated with the stromal keratocytes and extracellular matrix was measured by taking the area under the curve between the location of the basal lamina peak (top of the stroma) and the endothelial peak [34].

### 4.6. 3D Human Corneal Stromal Model

All human studies were performed with Institutional Review Board (IRB) approval from the University of Oklahoma Health Sciences Center (protocol #3450). Written and informed consent was obtained prior to tissue collection and processing. All methods were performed in accordance with federal and institutional guidelines, adhered to the tenets of the declaration of Helsinki, and human samples were de-identified prior to analysis. Primary human corneal fibroblasts were isolated from cadaveric corneas, as previously described [35,36]. To test the effects of quercetin on gene expression patterns, 1x10^6 human corneal fibroblasts per well (passage 4) were seeded into six-well polycarbonate transwell plates with 0.4 μm pores (Transwell; Corning Costar, Charlotte, NC) in complete fibroblast medium (Eagle’s Minimum Essential Medium (ATCC, Manassas, VA, USA), 10% fetal bovine serum (Atlanta Biologicals, Lawrenceville, GA, USA), and 1 × antibiotic–antimycotic solution (100 units/mL penicillin, 100 μg/mL streptomycin, and 250 ng/mL amphotericin); Gibco, Life Technologies, Grand Island, NY, USA) [19,20]. Following 24 h incubation, constructs were stimulated with a stable Vitamin C derivative (0.5 mM 2-O-α-d-glucopyranosyl-l-ascorbic acid (CAS #: 129499-78-1; American Custom Chemicals Corporation, San Diego, CA, USA) containing various concentrations of Quercetin solution (5 μm, 10 μm, and 15 μM). The constructs were supplied with fresh medium + treatments every other day for four weeks and were then subsequently processed for mRNA expression using quantitative real-time polymerase chain reaction (qRT-PCR) analysis. The four-week timepoint is consistent with previous studies from our group using this model to allow for stromal thickening and maturation [36,37].

### 4.7. Quantitative Real-Time Polymerase Chain Reaction (qRT-PCR)

Whole RNA extraction was performed on the human corneal fibroblast constructs (Ambion TRIzol^®^ Plus RNA Purification Kit: Life technologies, Carlsbad, CA, USA), followed by cDNA synthesis (SuperScript™ III First-Strand Synthesis SuperMix; Invitrogen; Carlsbad, CA, USA) using standard protocols. Taqman gene expression assays were used for housekeeping and target probes, which are listed in Table S1. The cDNA amplification was performed using TaqMan Fast Advanced Master Mix (Applied Biosystems, Foster City, CA, USA) incubated with TaqMan probes and 10 ng of cDNA using the QuantStudio™ 3 Real-Time PCR System (ThermoFisher Scientific; Rockford, IL, USA). The target genes that showed no detectable expression in any group were excluded from the analysis.

### 4.8. Statistical Analysis

All statistical analyses were performed in GraphPad Prism (version 9.1.1 for Windows, GraphPad Software, San Diego, CA, USA). A two-way analysis of variance (ANOVA) with Šídák’s multiple comparisons test was used to compare multiple timepoints and drug concentrations. For the rabbit haze analysis, since groups did not meet the equal variance assumption, a Brown–Forsythe ANOVA test and Dunnett’s T3 multiple comparison test were used. Ordinary one-way ANOVA analysis with Šídák’s multiple comparisons test was used to compare the different in-vitro quercetin treatments. A *p*-value of < 0.05 was considered statistically significant.

## 5. Conclusions

In a mouse debridement model, a single application of 5 mM quercetin eyedrops following corneal debridement led to a significant decrease in scar severity that was maintained for up to 3 weeks following injury compared to the vehicle treatment. In a LK rabbit model, quercetin appeared to reduce corneal haze in two of the three rabbits, but did not reach our threshold for statistical significance (*p* < 0.05) when compared to the vehicle-treated controls. Gene analysis of primary human corneal fibroblasts treated with quercetin in vitro showed a significant upregulation of SMAD7 and TGFB2.

## Data Availability

Data available upon request from corresponding author. Data available on request due to restrictions on privacy.

## Supplementary Materials

The following supporting information can be downloaded at: https://www.mdpi.com/article/10.3390/metabo12070626/s1. Table S1: qRT-PCR probes and catalog numbers; Figure S1: Slit lamp images of mouse eye at weeks 1, 2, and 3 following wounding; Figure S2: Hematoxylin and eosin (H&E) staining of an unwounded mouse eye isolated at week 3; Figure S3: H&E staining of a wounded mouse eye treated with 10% DMSO immediately following injury and isolated at week 3; Figure S4: H&E staining of wounded mouse eye treated with a 50 μM quercetin solution immediately following injury and isolated at week 3; Figure S5: Summary of findings in the animal models; Supplemental Methods: Histology. Reference [38] is cited in the supplementary materials.
