# Supplementary material for: Quercetin Decreases Corneal Haze In Vivo and Influences Gene Expression of TGF-Beta Mediators In Vitro
Source: Metabolites. 2022 Jul 7;12(7):626. doi: 10.3390/metabo12070626 (PMC9318747; doi:10.3390/metabo12070626)
Supplement: Supplementary file 1 [file metabolites-12-00626-s001.zip › metabolites-1768568-supplementary.pdf]

Supplemental Materials

Table S1.

Figure S1.

Figure S2.

Figure S3.

Figure S4.

Figure S5.

Supplemental Methods.

References.

Taqman assay name	Catalog ID
GAPDH	Hs99999905_m1
18S	Hs99999901_s1
COX2/PTGS2	Hs00153133_m1
CD34	Hs00990732_m1
DCN	Hs00370385_m1
ERK1/MAPK3	Hs00946872_m1
ERK2/MAPK1	Hs01046828_m1
ERK4/MAPK4	Hs00969401_m1
ERK5/MAPK7	Hs00964718_m1
HO-1/HMOX1	Hs01110250_m1
iNOS/ISYNA1	Hs01126940_gH
JNK1/MAPK8	Hs01548514_m1
JNK1/MAPK8	Hs01548508_m1
JNK2/MAPK9	Hs01558224_m1
Keratocan	Hs00559942_m1
LTBP1	Hs01558763_m1
LTBP2	Hs00166367_m1
LTBP3	Hs01005746_m1
LTBP4	Hs00943217_m1
MCT1/SLC16A1	Hs01560299_m1
MCT2/SLC16A7	Hs00940851_m1
MCT4/SLC16A3	Hs00358829_m1
NFκB	Hs00765730_m1
NRF2/NFE2L2	Hs00975960_m1
PI3Kα/PI3CA	Hs00907957_m1
p38/RPP38	Hs00946166_s1
p38delta/MAPK13	Hs00559622_m1
RHOA	Hs00357608_m1
SMAD2	Hs00183425_m1
SMAD3	Hs00969210_m1
SMAD4	Hs00929647_m1
SMAD6	Hs00178579_m1
SMAD7	Hs00998193_m1
TGFB1	Hs00998133_m1
TGFB2	Hs00234244_m1
TGFB3	Hs01086000_m1
TGFBRI	Hs00610320_m2
TGFBRII	Hs00234253_m1

Table S1. qRT-PCR probes and catalog numbers. (Manufacturer: ThermoFisher, Waltham, MA).

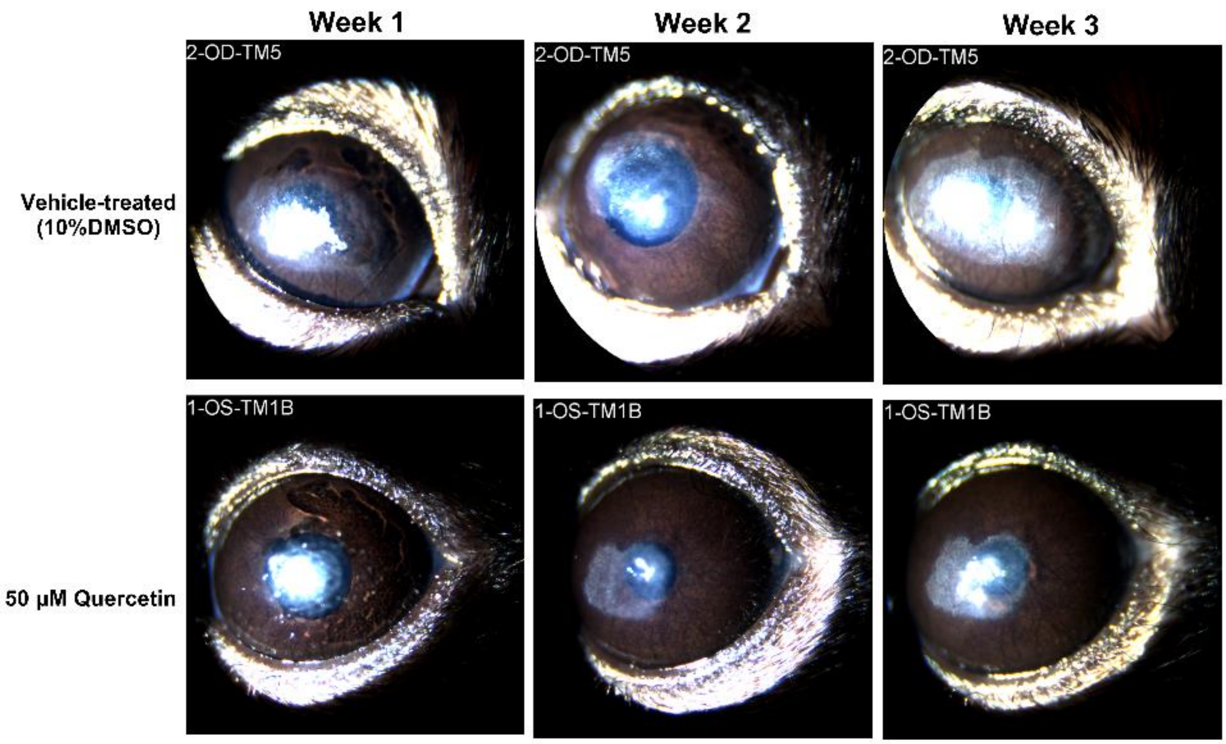

Figure S1. Slit lamp images of mouse eye at weeks 1, 2, and 3 following wounding. Low-dose quercetin solution (50 µM) in 10% DMSO (vehicle) were tested and topically applied to the cornea immediately post-debridement. Representative images shown for each treatment group (n=3 eyes for quercetin-treatment and n=3 eyes with vehicle-treatment).

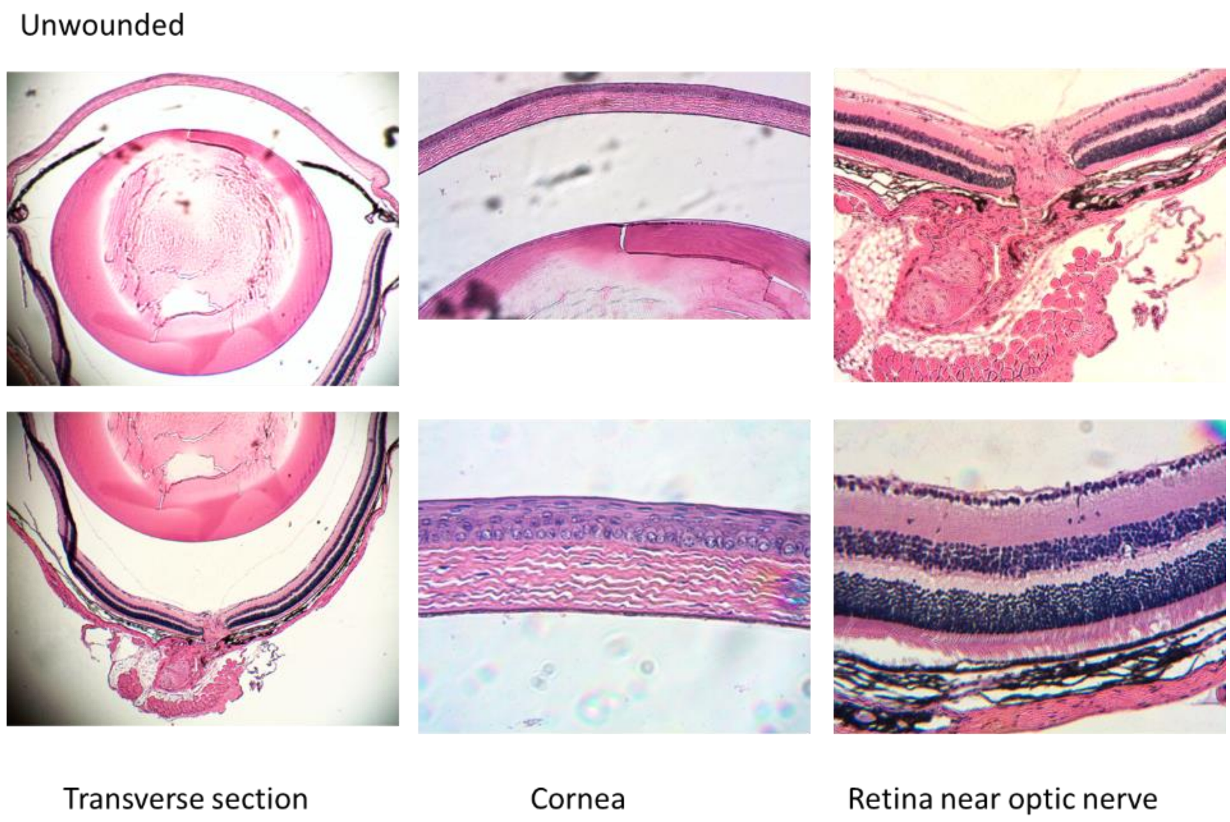

Figure S2. Hematoxylin and eosin (H&E) staining of an unwounded mouse eye isolated at week 3.

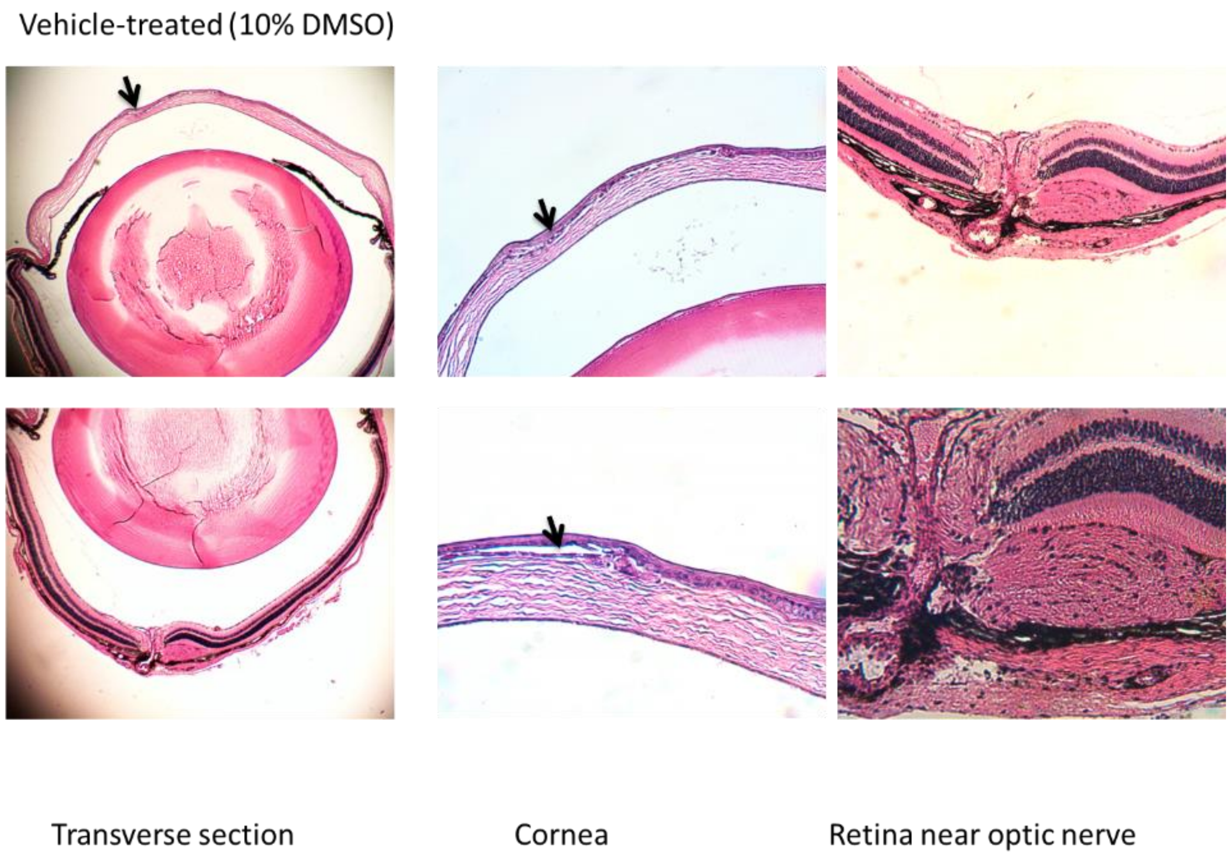

Figure S3. H&E staining of a wounded mouse eye treated with 10% DMSO immediately following injury and isolated at week 3.

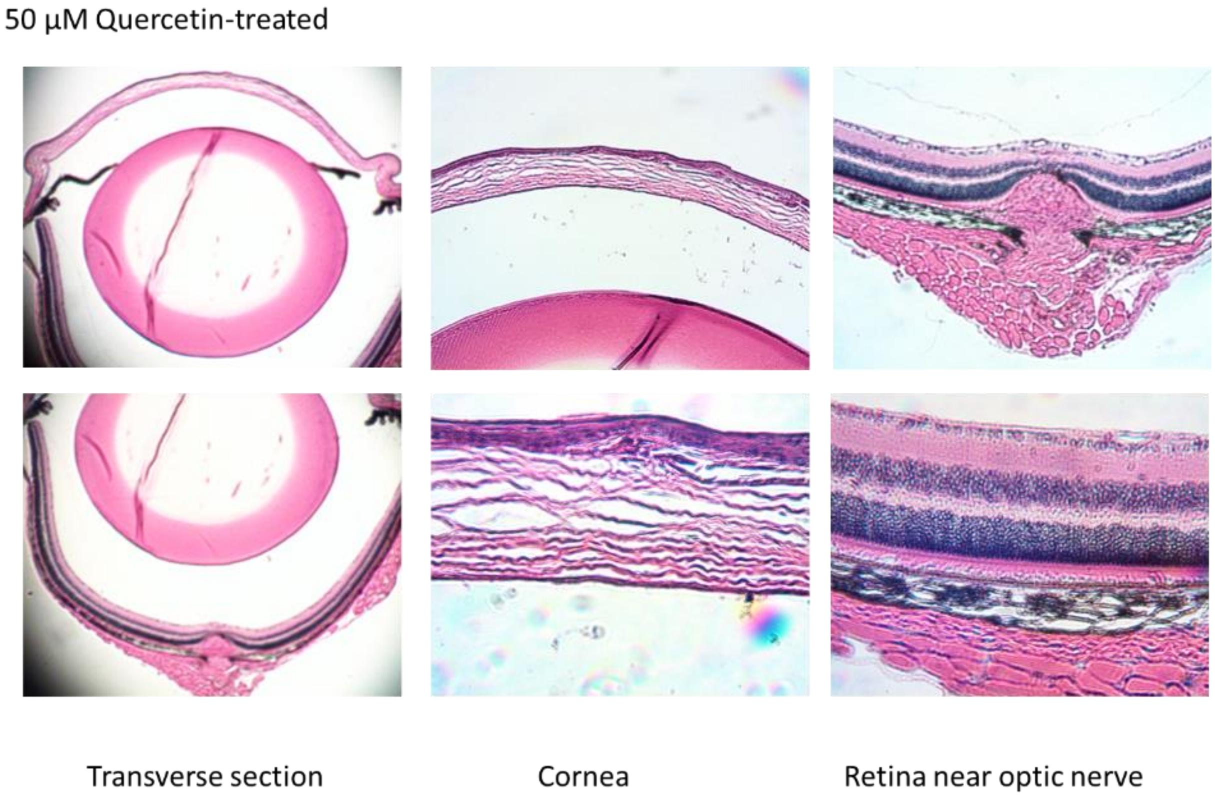

Figure S4. H&E staining of a wounded mouse eye treated with a 50 µM quercetin solution immediately following injury and isolated at week 3.

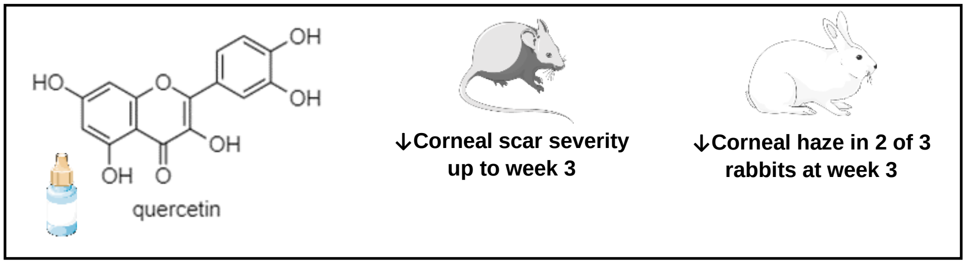

Figure S5. Summary of findings in the animal models. In mice, a single-topical application of 5 mM quercetin was performed following epithelial/stromal debridement. In rabbits, topical application of 5 mM quercetin was performed twice daily for 3 days following lamellar keratectomy. The pictorials were partly generated using Servier Medical Art, provided by Servier, licensed under a Creative Commons Attribution 3.0 unported license.

Supplemental Methods:

Histology

Mice were euthanized at week 3 by CO_2 inhalation followed by enucleation. Eyes were collected for histology analysis and immediately immersed in 5% paraformaldehyde in PBS and placed on ice. Samples were then processed for paraffin sectioning and stained with H&E using standard procedures.[1]

References:

1. Fischer, A. H., Jacobson, K. A., Rose, J., & Zeller, R. (2008). Hematoxylin and eosin staining of tissue and cell sections. CSH protocols, 2008, pdb.prot4986. https://doi.org/10.1101/pdb.prot4986.
